# Breaking Bad News Issues: A Survey Among Radiation Oncologists

**DOI:** 10.4103/0973-1075.53533

**Published:** 2009

**Authors:** Milind Kumar, Shikha Goyal, Karuna Singh, Subhas Pandit, DN Sharma, Arun K Verma, GK Rath, Sushma Bhatnagar

**Affiliations:** 1 Department of Radiotherapy, Dr. BR Ambedkar Institute Rotary Cancer Hospital, All India Institute of Medical Sciences, New Delhi - 110 029, India; 2 Department of Anaesthesia and Palliative Care, Dr. BR Ambedkar Institute Rotary Cancer Hospital, All India Institute of Medical Sciences, New Delhi - 110 029, India

**Keywords:** Bad news, Cancer, Radiation oncology

## Abstract

**Introduction:**

Discussion of bad news and resuscitation in terminal cancer is an important but difficult and often neglected issue in day-to-day oncology practice.

**Materials and Methods:**

We interviewed 35 radiation oncologists using an indigenous 15-item questionnaire on their beliefs about breaking bad news and resuscitation to terminal cancer patients.

**Results:**

Most responders had an oncology experience of three to seven years (20/35). Thirty-two were comfortable discussing cancer diagnosis, prognosis and life expectancy-related issues. A similar number believed all cancer-related information should be disclosed, while only four believed in imparting all information in one visit. All agreed that disclosing sensitive information did not affect survival. When requested by relatives to withhold truth from patients, 11 said they would not comply, 22 agreed to tell the truth only if asked and two agreed to avoid difficult questions. Twenty responders denied having been adequately trained in breaking bad news and were keen on dedicated classes or sessions in this area of practice. Most (33/35) believed that Indian patients were keen on knowing their diagnosis and prognosis. Although all agreed to the importance of discussing resuscitation, only 17 believed patients should be involved. Majority (20/35) agreed that the issue needs to be discussed while the patient was conscious. Patients with unsalvageable disease were deemed unsuitable for aggressive resuscitation by 30 responders while the rest believed it should be offered to all. Most (21/35) admitted to feeling depressed after breaking bad news though only seven felt disclosure was more stressful than untruthful statements. Only four knew of a law regarding resuscitation in cancer.

**Conclusion:**

Observing the widely varied beliefs and practices for disclosing bad news, it is recommended that such training be a regular part of medicine curriculum, especially in the Oncology setting.

## INTRODUCTION

Aspects of breaking bad news in our culture has been relatively less discussed due to stigma and also the widespread belief that if a person comes to know about cancer diagnosis, the life expectancy of that person falls dramatically. There are no guidelines or academic teaching as to how we differ from the Western countries in such aspects.

In Western countries most of the issues are patient-led and family takes a supportive role. In Eastern cultures such as India, patient's illness is considered a family event and the family remains central in many decision making and at times may demand exclusion of the patient from decision-making for which there is no uniform consensus amongst health care providers.

With these issues in mind, a study was conducted amongst the radiation oncologists in our teaching hospital and a survey was done to assess the beliefs in treating radiation oncologists regarding this vital aspect of cancer care.

## MATERIALS AND METHODS

**Figure 1:**
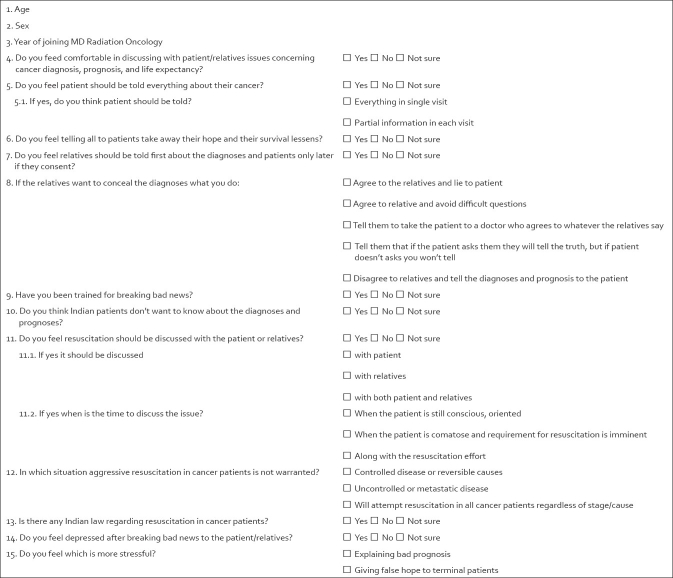
Questionnaire used in the study

A 15-item questionnaire was developed to assess two aspects of cancer care amongst radiation oncologists: issues pertaining to disclosing diagnoses and breaking bad news (Q. 4-10, 14, 15), and knowledge and attitude regarding resuscitation of terminal cancer patients (Q. 11-13) [Figure 1]. Age, gender and experience in oncology of the respondents were also recorded. Radiation oncologists from the Department of Radiotherapy at a tertiary care teaching hospital were administered this questionnaire and their responses evaluated.

## RESULTS

A total of 35 radiation oncologists participated in the survey. Anonymity was maintained during analysis of the results. A majority of the responders were senior residents with experience from three to around seven years (including non-teaching hospital experience) [Table 1].

**Table 1:** Experience of respondents in oncology

Designation	Duration of experience	Number surveyed
Under training (MD Radiotherapy)	6 months to 3 years	6
Post MD SR ship	3 to 7 years	20
Consultants (including research/pool officers)	7 to 28 years	9

Table 2 lists the questions and responses pertaining to breaking bad news to cancer patients.

**Table 2:** Responses to queries on disclosing diagnosis, prognosis in cancer patients

Questions	Responses (Total = 35)
Q4. Do you feel comfortable discussing with patient/relatives issues concerning cancer diagnosis, prognosis, and life expectancy?	Yes: 32
	No: 3
Q5. Do you feel patient should be told all about their cancer?	Yes: 32
	No: 2
	Not sure: 1
Q5.1. If yes, do you think patient should be told?	Everything in Single visit: 4
	Partial information in each visit: 31
Q6. Do you feel telling all to patients takes away their hope and lessens survival?	No: 35
Q7. Do you feel relatives should be told first about the diagnoses and patients only later if they consent?	No: 35
Q8. If relatives want to conceal the diagnoses what do you do?	Agree to relative and avoid difficult questions: 2
	Tell them that if the patient asks them they will tell the truth, but if patient doesn't asks you won't tell: 22
	Disagree to relatives and tell the diagnoses and prognosis to the patient: 11
Q9. Have you been trained to break bad news?	Yes: 15
	No: 20
Q10. Do you think Indian patients don't want to know about the diagnoses and prognoses?	Yes: 1
	No: 33
	Not sure: 1
Q14. Do you feel depressed after breaking bad news to the patient/relatives?	Yes: 21
	No: 14
Q15: Which is more stressful?	Breaking bad news 7
	Giving false hope 28

The issue of comfort level during breaking bad news was asked, to assess the level of self awareness of the treating physician with regards to these difficult issues. A good 91.4% thought they were comfortable discussing these issues. There was broad agreement (91.4%) that patients be told all about their illness. However, only 11.4% believed that all information should be given in a single visit; all others felt that partial information be given over multiple visits, probably to aid in comprehension and improve acceptance to such information. Out of four responders for complete information, one answered that it would depend on patient's understanding and receptivity hence presuming the patient understands, he would tell all in a single visit. None supported the belief that disclosure of cancer diagnosis adversely impacts survival, contrary to popular myth. Only 31.4% felt they would disclose the diagnosis to patients even against the wishes of relatives. Majority (62.9%) felt they would give complete information to the patient only on being asked. None said they would agree to the wishes of relatives to conceal diagnosis under all circumstances. Twenty (57%) of the respondents felt they would benefit from further training in this aspect of breaking bad news. Contrary to general feeling, 94.3% of respondents believed that Indian patients are keen on knowing of their disease. Most (60%) respondents agreed that they felt depressed after disclosing bad news to patients. However, a good number (40%) did not report having any such feelings. Interestingly, 80% considered giving false hope more stressful than disclosure of diagnosis.

Table 3 lists the responses to questions related to resuscitation in terminal patients. All respondents realized the importance of discussing the issue of resuscitation with patients and relatives. However, only 48.5% believed that patients should be involved in this discussion. Most (80%) believed that the best time for such discussions would be when the patient is conscious and oriented; 20% believed this discussion should take place only when the patients is comatose and requirement of resuscitative measures in imminent. Only 14.3% believed that aggressive resuscitation should be offered to all irrespective of stage or cause, the rest believed it should be discouraged in uncontrolled or metastatic disease. Only 11.4% were aware that there was an Indian law addressing the issue of resuscitation in terminal patients.

**Table 3:** Responses to questions addressing issues pertaining to resuscitation in terminal cancer patients

Questions	Responses (Total= 35)
Q 11. Do you feel resuscitation should be discussed with the patient or relative?	Yes: 35
Q 11.1 If yes it should be discussed with	With relatives 15
	With both patient and relative 17
Q11.2 If yes when is the time to discuss the issue?	When the patient is still conscious, oriented 28
	When the patient is comatose and requirement for resuscitation is imminent 7
Q 12. In which situation aggressive resuscitation in cancer patients is not warranted?	Uncontrolled or metastatic disease 30
	Will attempt resuscitation in all cancer patients regardless of stage/cause. 5
Q 13. Is there any Indian law regarding resuscitation in cancer patients?	Yes: 4
	No: 15
	Not sure: 16

## DISCUSSION

Bad news may be defined as “any information which adversely and seriously affects an individual's view of his or her future”.[1] Breaking bad news to patients and families is a frequent, essential and stressful task for the physicians, especially for those dealing with cancer. Communication of bad news has been a topic of dilemma since the early history of medicine, with accounts of Hippocrates recommending hiding of any information that could cause despair and worsen the patient's situation. This notion was confirmed in the first ethical code in medicine in 1847 that guided doctors not to disclose bad news to patients due to possibility of shortening their lifespan. In many Asian cultures, it is considered unnecessarily cruel to directly inform a patient of a cancer diagnosis.[2] Consequently, this approach of preventing harm by keeping the patient ignorant of their condition became a common practice worldwide. However, the notion that receiving such unfavorable information will invariably be psychologically detrimental has never been substantiated.[3]

Most patients like to receive accurate information that helps them make important decisions that have a bearing on their future plans and quality of life. Several studies in varied communities have shown that a large proportion (over 90%) of patients are keen on knowing if they had cancer, getting a realistic estimate of survival as well as expected benefits and adverse effects with available therapy[4,5] A study at MD Anderson endeavored to assess cancer patients' preferences for disclosure of unfavorable news. A total of 351 patients responding to a 46-item questionnaire, gave a high rating to physician expertise, discussion of options, clarity and honesty with which the information is imparted, while supportive aspects of communication such as comforting were given slightly less importance.[6] Another study indicated that patients were more satisfied after discussions with their family physicians rather than oncologists, and suggested that oncologists should involve family physicians in disclosing bad news to patients.[7]

The comfort level with bad news discussions, in our study, was higher than in the aforementioned studies, probably because of a tertiary cancer hospital setting and the participants being highly sensitized to the issue owing to the patient characteristics including advanced disease routinely encountered in daily clinical practice. However, a large majority (60%) admitted to feeling depressed after such disclosure, showing the task of explaining diagnosis and prognosis is not simple and can cause temperament changes and work dissatisfaction. There was broad agreement that patients should know of their disease in conformity with international guidelines. Disclosing all the information in one visit may be too much information to comprehend for a majority of patients. Giving partial information remains the best strategy, since it has been observed that patients retain less than a quarter of the information imparted to them in one session. It is a common concept in India that the patients do not want to know about their disease. The fact that 33/35 respondents felt that Indian patients would like to know about their disease reflects the change in Indian society that in the past times, the doctors were supposed to decide the fate of the patients, but now the patient want to know their disease and take active part in decision making.

Most people still tend to view a diagnosis of cancer as an imminent death sentence. The responsibility of striking the difficult balance between alarming patients unnecessarily and falsely re-assuring them largely rests with the treating physician. Disclosing to patients that they have cancer is an accepted practice now in western cultures and there is evidence in support of frank discussion about diagnosis and treatment details with cancer patients.[8]

A 35-item questionnaire administered to doctors, nurses and patients showed that caregivers and patients both gave similar importance to presence of family members during the “bad news” interview. Patients gave more importance than caregivers to disclosure of bad news as close as possible to diagnosis, and to discussion of therapy and possible adverse effects. There was general agreement on the subject that patient, and not the caregiver, will decide on the amount of information to be delivered. The authors found that participatory and encouraging sentences were more important to facilitating communication and confidence, and advocated avoidance of hopeless sentences. Predominant caregiver emotions included a sense of identification with the patient followed by a feeling of hopelessness. Most caregivers were not qualified on the issue of breaking bad news.[9]

An important ethical principle is that clinical information should be discussed with a patient first and with relatives only if the patient has given permission for the doctor to do so. The doctor needs to be assertive whilst remaining approachable and re-assuring. He needs to explain to the relatives the ethical position as well as the potential difficulties caused by keeping patients ignorant of their diagnosis such as confusion and loss of confidence, reassuring them about carrying out his discussion with the patient in as sensitive a way as possible and that the patient's desire of the extent to which the information is given would be respected. Relatives may be informed that being upset with a cancer diagnosis is a natural response but many people do adjust to that knowledge.[10] None of the respondents in the current study supported the notion of limitation of life expectancy with unfavorable diagnosis disclosure, held by relatives of most cancer patients who confront the doctor to not to tell bad news otherwise the patient may be heartbroken and will die earlier or lose the will to live. One of the most difficult scenarios frequently seen in clinical practice is when the relatives demand that the patient should not be told the diagnoses. We observed in our study that a vast majority (33/35) was keen on truth-telling, though most (22/35) agreed to respect their wishes to the extent that they would not volunteer such information themselves, but would convey it if asked. The ideology of disclosing all to the patients regardless of relatives not agreeing is difficult to implement in a busy hospital setting and many a times the doctors accept the strategy of concealment by the primary care giver due to paucity of time or other reasons. Illness is considered a family event rather than an individual occurrence. Hence, family based medical decisions are taken in preference to individual wishes.[11]

A survey in Italy showed that only 44% of physicians would inform patients of the cancer diagnosis and their prognosis if the patient wanted to know but the family members were opposed to informing the patient.[12] Another physician survey revealed that only 44.8% believed that cancer patients should always be informed of their diagnosis, another 46.6% believed that patients should be told the truth ‘only in some cases’. In practice, only 25% actually disclosed the diagnosis. Nearly a third of responders believed that patients never want to know the truth. A large majority (86%) felt the need of having guidelines for breaking bad news to patients.[13] Nearly 57% respondents in our study felt they would benefit from further training in dealing with such issues, indicating the deficit in care even in highly specialized settings.

An informal survey conducted at 1998 annual meeting of American Society of Clinical Oncology revealed that over 60% respondents broke bad news to patients 5-20 times in a month, and another 14% over 20 times a month. Over 55% rated the process of being honest without taking away hope as the most difficult task, while 25% considered dealing with patients' emotions as the most difficult. Fewer than 10% of the respondents had received any formal training in breaking bad news, and nearly half rated their communication skills as poor to fair. Baile and colleagues from MD Anderson Cancer Center proposed a 6-step protocol (SPIKES) for disclosure of bad news: setting up the interview, assessment of patients' perception, obtaining patient's invitation for degree of detail desired, giving knowledge and information to the patient, addressing patient's emotions with emphatic responses, and devising a strategy for future management in collaboration with the patient along with summarizing the discussion to avoid misinformation.[14]

An interview of 30 Swedish physicians with experience in medicine ranging from 1-21 years indicated that 30% had no training in breaking bad news and 87% perceived this task as difficult, at least occasionally despite 90% of all having to give such information to patients over five times in the previous year. Breaking bad news was perceived as involving a risk of losing control in different ways, regarding emotions, oneself, confidence, professionalism and patient trust.[15]

Unlike the earlier belief that communication skills can improve solely with years of medical practice, it is now well established that the principles of communication skills can be delivered through didactic means such as tutorials, lectures, textbooks, and other aides (CD-ROMS, web sites), which may be refined through practice and experience. Role play has emerged as an effective technique despite the initial reluctance of participants. Most medical schools in the West are incorporating communication skills training in the undergraduate and postgraduate curriculum in order to better prepare the budding doctors for their role of efficient communicators. Communication skill workshops for practicing physicians are becoming a regular feature of annual meetings of many cancer societies.[16]

Resuscitation remains a tricky and difficult area to discuss with the patient and relatives. One of the most important issues in cancer care is as to which are the cancer patients in whom resuscitation should be offered. Over 85% respondents in our study felt that resuscitation should only be done in patients with controlled primary or those with acute and reversible causes. The information about any law for resuscitation in India remains very low in treating doctors. This goes on to reflect the low level of legal awareness regarding such an issue which affects the oncology practitioner on a day to day basis. The Indian Society of Critical Care Medicine Guidelines state that pending consensus decisions or in the event of conflicts between physician's approach and the family's wishes, all existing supportive interventions should continue.[17]

## CONCLUSION

Our findings confirm that the treating oncologists are keen on sharing information with their patients, since they believe that Indian patients are interested in knowing their diagnoses and prognosis. Even though the oncologists feel they are comfortable with breaking bad news, more than half feel they need more training and feel depressed after the same. The relatives' attitudes and beliefs versus patient's wishes remains a tricky area especially in Indian social context. Resuscitation remains a difficult area with disagreements on patient's involvem ent and final decision making, with legal awareness wanting.

There is an urgent need for incorporation of breaking bad news in medical curriculum along with the contemporary legality issues in cancer patients. Observing the widely varied beliefs and practices for disclosing bad news, it is recommended that such training be a regular part of medicine curriculum, especially in the oncology setting. Availability of guidelines or recommendations from professional organizations like Indian Association of Palliative Care or Association of Radiation Oncologists of India in this regard would go a long way in resolving some of these issues to a large extent.
